# The Journey of Mango: How the Shipping Systems Affect Fruit Quality, Consumer Acceptance, and Environmental Impact

**DOI:** 10.3390/plants14213241

**Published:** 2025-10-22

**Authors:** Cosimo Taiti, Bruno Bighignoli, Giulia Mozzo, Elettra Marone, Elisa Masi, Diego Comparini, Edgardo Giordani

**Affiliations:** 1 Department of Agricultural, Food, Environmental and Forestry Sciences and Technologies (DAGRI), University of Florence, Viale delle Idee 30, 50019 Sesto Fiorentino, Italy; cosimo.taiti@unifi.it (C.T.); giulia.mozzo@unifi.it (G.M.); elisa.masi@unifi.it (E.M.); diego.comparini@unifi.it (D.C.); edgardo.giordani@unifi.it (E.G.); 2 Faculty of Biosciences and Technologies for Agriculture, Food and Environment, University of Teramo, Via R. Balzarini, 1, 64100 Teramo, Italy; emarone@unite.it

**Keywords:** mango fruit, shipping methods, consumer and sensory evaluations, PLS-DA, VOCs profiling, GHG emissions

## Abstract

Mango (*Mangifera indica* L.) is a popular tropical fruit enjoyed worldwide, with Europe being a significant importer of this fruit. Its climacteric nature and short shelf-life pose challenges for maintaining quality, while emissions from transportation threaten the sustainability of the supply chain. This highlights the importance of low-impact logistics in maintaining fruit quality. This study aimed to evaluate the quality of fresh mangoes in Italy by comparing the different shipping systems (air, sea, and road) for seven cultivars sourced from seven countries. Quality assessment included pomological analysis, PTR-ToF-MS for volatile profiling (*n* = 11 cultivars × 2 years × 3 replicates), and consumer sensory analysis (*n* = 65 for untrained panellists in 1 year, *n* = 8 for trained panellists over 2 years). Results indicated that air and truck transport better preserved fruit quality compared to sea freight, primarily due to shorter transit times, which allowed for harvesting at more advanced ripeness stages. The combination of PTR-ToF-MS and PLS-DA effectively differentiated samples based on the method of transport, showcasing its potential as a quick quality monitoring tool. Mangoes transported by air showed significantly higher levels of volatile organic compounds (VOCs), a 29% greater total soluble solids (TSSs) content, and a 44% lower acidity (TA). Sensorial tests indicated that consumers preferred these mangoes. However, air transport resulted in 30 times higher CO_2_ emissions per kg of fruit compared to sea freight (~642,117 CO_2_e (kg) vs. ~19,132 CO_2_e (kg)), highlighting a critical dilemma between sustainability and quality. These findings provide a framework for developing hybrid logistics strategies that strike a balance between preserving quality and environmental responsibility. Additionally, they support the development of European mango cultivation, which can optimise harvest timing, reduce emissions, and enhance fruit quality.

## 1. Introduction

Exports of fresh fruits from tropical countries have steadily increased over the past few decades; however, limited consumer familiarity with these products has hindered their introduction into global markets [1]. Mango (*Mangifera indica* L.) is one of the most widely consumed tropical fruits in the world, serving as an important cash crop for many producing countries [1,2]. Global demand for tropical fruits is steadily increasing, with Europe and the United States identified as the largest import markets [3,4,5]. In Europe, consumption growth reflects an interest in aesthetic appeal, vibrant colours, and unique flavours [2,6].

Despite this, the availability of tropical fruits remains limited, suggesting a strong potential to expand the availability of fresh exotic products [7,8]. Consumers are drawn to their visual appeal, aroma, and flavour, as well as growing awareness of their nutritional benefits and role in supporting overall health [9]. However, perceived health benefits alone do not significantly motivate purchases of tropical fruits [10]. Without a satisfying sensory experience, especially given higher prices than traditional fruits, consumers may abandon them after initial trials [11].

Economic and environmental aspects are increasingly relevant. In Thailand, sustainability assessments reveal that while the mango value chain provides substantial economic benefits, it also poses significant environmental challenges, including water and carbon footprints [12]. Brazilian mango production in semiarid regions similarly highlights the water and carbon impacts of cultivation [13].

Mango’s short shelf life, which rarely exceeds three weeks even under ideal storage conditions, makes harvest timing, storage, and transport critical for quality [14,15,16]. Harvest generally occurs at physiological maturity (mature but not fully ripe) to withstand handling and transport. Premature harvest can result in immature fruits that do not complete ripening, which can significantly impact the final quality of the product [17,18].

Flavour and aroma are primarily determined by volatile organic compounds (VOCs) produced during ripening, which serve as key quality indicators valued in the European market [19,20]. Ripening yields a complex blend of VOCs that shape flavour perception [21], with each cultivar displaying a unique VOC profile that changes daily [22]. These compounds are influenced by cultivar and environmental conditions during ripening and postharvest conditions, including transport [23].

Transport mode influences harvest timing: air-freighted mangoes are typically picked at near-optimal ripeness due to their short transit times; maritime shipments, on the other hand, require an earlier harvest to survive longer journeys [24,25,26]. Truck transport results in intermediate journey time and uses intermediate maturity (70–85% ripeness) [17,27]. Additionally, storage conditions at the time of harvesting and during transportation can significantly impact the quality of the final product. Postharvest technologies, such as refrigeration, controlled/modified atmosphere, and ethylene gas scavenging materials, can delay fruit ripening, thereby maintaining quality during storage and transport [28,29]. Modal choice is thus critical for both carbon footprint and quality outcomes. Life Cycle Assessments must integrate these disparities, as emission intensities vary widely. Air transport allows full maturity but has 30–60 times the environmental impact of maritime shipping [30].

Port-to-port analyses can underestimate total impact, as pre- and post-haulage stages account for over 40% of total transport-related emissions [31,32]. Door-to-door evaluations capture the whole logistic chain, addressing a key gap in conventional food LCAs [33]. Transport also imposes physiological trade-offs: air freight preserves quality through near-ripe harvesting, while maritime shipping risks flavour loss and higher spoilage due to premature harvesting. These effects remain largely absent from current regulations and sustainability assessments, underscoring the need for integrated approaches that address both environmental and quality considerations [34].

A comprehensive quality assessment based on sensory perceptions (visual, olfactory, gustatory) can guide strategies in the international value chain and prevent negative market experiences [35]. Measuring VOC composition can effectively assess quality throughout the shelf life. Growing conditions also influence quality: research on imported mangoes under Mediterranean subtropical climates found significant differences in flavour, aroma, and consumer acceptance [36], emphasising the importance of cultivar and cultivation environment in shaping quality and preference.

European consumers have consistently valued aroma and sweetness in fruits, favouring premium-quality products [37]. Growing concerns over food miles have led to an increase in mango cultivation in Mediterranean regions [38]. Spain produces over 40,000 tons annually (FAO, 2021) [39], and Italy has expanded mango farming in southern regions, such as Sicily and Calabria, primarily for export. Sicily’s humid winter and hot, dry summer allow a harvest window of up to six months (June–November) [37,40]. This local supply aligns with the European market’s preference for tree-ripened fruit, enabling delivery within 24–48 h after harvest and improving freshness while reducing the carbon footprint. Despite the potential, data on the quality, nutraceutical value, and sensory attributes of Italian mango farmers remain scarce. Given the importance of sensory experience in European consumption of tropical fruits [41] and the rising demand for high-quality produce, evaluating factors such as post-harvest handling, transport, and sustainable production is essential. Consumers often find imported mangoes to be under-ripe, bland, or lacking sufficient sweetness [42]. Nevertheless, consumer studies in major importing countries are limited [4,43], reflecting the fact that only about 4% of global mango production is exported to markets, with most fruit consumed in the countries of production [44]. Consequently, existing research on consumer perceptions has focused mainly on consumers in producing regions, particularly in developing nations [45,46,47].

**Figure 1:**
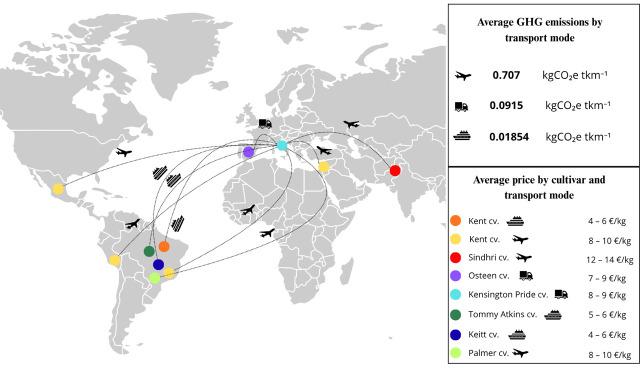
Trade routes for mango imports from various exporting countries to Florence, showing the transport modes used, their associated average greenhouse gas emissions (kg CO_2_e tkm^−1^), and the average price of each mango cultivar according to the transport method.

This study aims to analyse the current availability of fresh mango in Tuscany and evaluates the quality standards of the main cultivated varieties. Figure 1 illustrates the import routes from major exporting countries to Florence. The specific objectives of this research were to (1) assess the current availability of fresh mangoes in Tuscany by comparing different “ready-to-eat” mango cultivars, using pomological parameters, volatile compound profiling, and sensory analysis; (2) identify the most effective shipping system to preserve high fruit quality and assessing their carbon footprint; and (3) determine the key quality factors influencing consumer liking, to understand consumer preferences for mangoes better.

## 2. Results

### 2.1. Chemical-Physical Characterisation of Mango Fruits

In the present study, variations in pomological characteristics were evaluated among seven mango cultivars (i.e., Kent, Kensington Pride, Keitt, Osteen, Palmer, Sindhri, and Tommy Atkins), which arrived on the Italian market via three different transportation methods (i.e., air, sea, and road). The study was conducted over two consecutive years in September to ensure consistency in the results. The characteristics of these cultivars are described in Table 1.

#### 2.1.1. Weight and Flesh Firmness

Table 1 presents the mean values and standard deviations (SDs) of sample weight across different mango cultivars, countries of origin, and transportation methods. Sample weights ranged from 578 ± 31 g for the Kent cultivar, which was shipped by air from Mexico, to 282 ± 23 g for the Sindhri cultivar, transported by air from Pakistan. Among the cultivars analysed, Tommy Atkins, originating from Brazil and shipped by sea, also exhibited a relatively high average weight (557 ± 33 g). In terms of fruit firmness, a distinct pattern emerged between samples transported by sea and those transported by air or road (Table 1). Specifically, fruits subjected to longer transportation times (sea transport) showed significantly higher pulp firmness, suggesting that extended transit durations, which require earlier harvest and longer storage periods, affect this attribute, as also reported elsewhere [23,26,49,50]. Average firmness values ranged from 2.73 ± 0.12 kg/cm^2^ for Tommy Atkins (sea transport from Brazil) to 0.74 ± 0.18 kg/cm^2^ for Sindhri (air transport from Pakistan). These findings suggest that transportation methods have a significant influence on fruit firmness, likely due to variations in harvest maturity and post-harvest handling practices required for market delivery.

#### 2.1.2. Total Soluble Solids (TSSs), Titratable Acidity (TA), Their Ratio (TSS/TA), and pH

Other key factors influencing mango quality include chemical parameters such as pH, total soluble solids (TSSs), titratable acidity (TA), and their ratio (TSS/TA). As a climacteric fruit, mango ripens postharvest, with complex carbohydrates breaking down to increase TSS due to sugar accumulation, while TA decreases as organic acids are metabolised [51]. The balance between sugars and acids is crucial for mango flavour and palatability, making the TSS/TA ratio a key indicator of fruit-eating quality and consumer acceptance [52]. As shown in Table 1, TSS ranged from a minimum of 12.50 ± 0.52 in the Tommy Atkins cultivar (from Brazil by boat) to a maximum of 18.75 ± 1.11 in the Sindhri cultivar (from Pakistan by plane). In line with Gentile et al. (2019) [40], TSS values varied significantly among cultivars. Notably, the Kent cultivars from different origins exhibited considerable variation in TSS values, primarily attributable to the transportation method: fruits that arrived by plane showed both higher TSS values and a higher TSS/TA ratio than those transported by boat. Thus, our data revealed that the TSS content and TSS/TA ratio were significantly higher in samples transported by air compared to those shipped by sea or road. The Kensington Pride cv as well as the Osteen cv reported an intermediate value in both TSS and TA, resulting in an intermediate TSS/TA ratio compared to the other mango cultivars. Both cultivars arrived on the Italian market from European countries by truck. TA content (meq/kg) was significantly higher in mango samples transported by boat (4.29 ± 0.43), followed by those transported by truck (3.42 ± 0.35) and plane (2.41 ± 0.81). Among the different cultivars, TA values ranged from a maximum of 4.63 ± 0.23 meq/kg in the Tommy Atkins to a minimum of 1.46 ± 0.60 meq/kg in the Sindhri cultivar, which arrived by air from Pakistan. Sindhri samples exhibited the highest TSS/TA ratio (12.80 ± 1.85), due to their high sugar content and low acidity, followed by the Kent cultivar from Mexico, which showed a high TSS/TA ratio (10.47 ± 1.40), primarily due to its low TA content. In contrast, Tommy Atkins exhibited the lowest ratio (2.70 ± 0.21), primarily due to its lower sugar concentration. Thus, like TA content, the mean ripening index (TSS/TA ratio) showed statistically significant differences based on the shipping method. pH values, when combined with TA data, provide a more accurate measure of fruit acidity, offering a fuller understanding of the fruit’s acid profile. As shown in Table 1, Tommy Atkins had the lowest pH value (3.54 ± 0.42), while the Sindhri cultivar from Pakistan exhibited the highest (5.43 ± 0.35). For the Kent cultivar, pH values varied more significantly with transportation method than with country of origin. Specifically, the Kent samples transported by sea from Brazil had an average pH of 3.73 ± 0.23, whereas those transported by air exhibited higher pH values.

### 2.2. VOCs Profiling by PTR-ToF-MS

This section presents the results of the PTR-ToF-MS analysis, which was employed to detect the volatile organic compounds (VOCs) emitted by the different mango samples. Since some compounds were detected only in trace amounts, it was not possible to accurately quantify their emission rate, and therefore, they were excluded from further analysis. Table 2 summarises the 41 putatively identified VOCs, based on their measured mass-to-charge (*m*/*z*) ratios, chemical formulas, and average concentrations ± standard deviation calculated over two years of trials.

**Figure 2:**
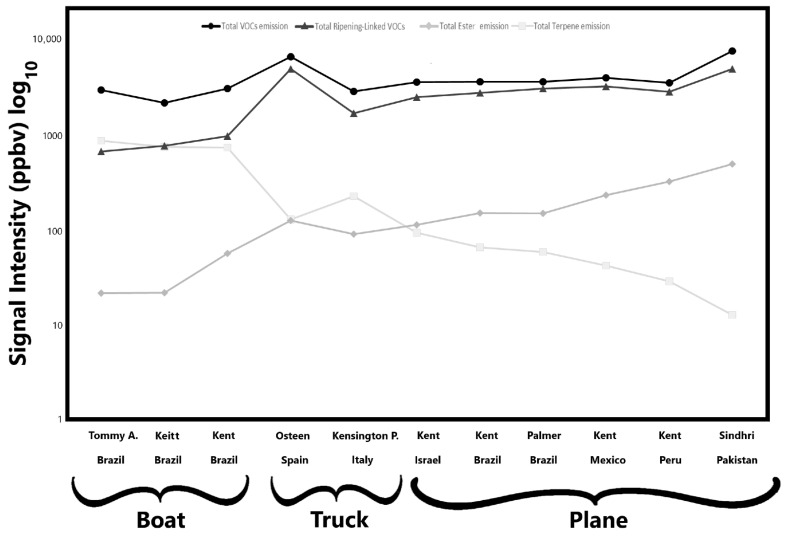
Mean signal intensities of five volatile organic compound (VOC) clusters detected in the mango samples. The figure illustrates the total emission levels of key VOC groups associated with each mango sample. VOC clusters were defined as follows: total VOCs (sum of all detected VOCs), total terpenes (sum of *m*/*z* 67, 81, 91, 93, 95, 109, 121, 123, 135, 137), total esters (sum of *m*/*z* 89, 103, 117), and ripening-associated VOCs (sum of *m*/*z* 33, 45, 47).

The data are organised by mango cultivar and transportation method. The analysis was performed on fresh pulp cubes obtained from peeled mangoes. Of these 41 detected signals, compounds belonging to alcohols, aldehydes, esters, ketones, hydrocarbons, and terpenes are present, while other signals reported are their fragments. As shown in Table 2, notable differences in VOC emission profiles were observed among the analysed samples, both in qualitative composition and emission intensity. Most of the reported compounds had been previously identified as standard components and/or key aroma compounds in mango [22]. Among these, esters are particularly significant, as they significantly contribute to the characteristic fruity aroma of mango [53]. To reduce dataset dimensionality and facilitate the interpretation of the volatile data, five VOC clusters are presented in Figure 2, which illustrates the total emission levels of key VOC groups (total VOC, terpenes, esters, and ripening-associated compounds) for each mango sample studied. Regarding total VOC emissions, no substantial differences were observed among cultivars, except for the Sindhri and Osteen cultivars, which exhibited the highest total emission levels, approximately 7650 ppbv and 6650 ppbv, respectively. Conversely, the Keitt cultivar, transported by sea from Brazil, showed the lowest signal intensity, at around 2150 ppbv. Moreover, no significant differences in total VOC emission levels were evident among the shipping methods. In general, as reported in Table 2, the highest VOC signal intensities were detected at *m*/*z* 45 (Tentatively identified as acetaldehyde), *m*/*z* 81 (TI C6 and terpene fragments), and *m*/*z* 33 (TI methanol). Notably, mangoes transported by air exhibited higher emission intensities of signal detected at *m*/*z* 45 and 33, suggesting increased levels of acetaldehyde and methanol, which are compounds typically associated with advanced ripening stages [54]. In contrast, fruits shipped by sea exhibited elevated signals at *m*/*z* 81, indicating a greater presence of C6 and terpene-related compounds, which are commonly associated with less advanced stages of ripening [23]. Similarly, when considering total ripening-linked VOCs, the Sindhri and Osteen cultivars again reported the highest values, nearly ten times greater than those observed in the Tommy Atkins cultivar (Table 2, Figure 2). In this case, however, apparent differences related to transportation methods emerged: samples arriving in the Italian market by boat showed lower levels of ripening-associated volatile compounds. In contrast, those transported by other methods exhibited higher concentrations, suggesting a more advanced ripening stage. On the contrary, a different trend emerges when considering total terpene emissions. As shown in Figure 2, a clear distinction can be made between samples transported by sea and those arriving by land, either by truck or plane. Mangoes shipped by boat exhibited significantly higher total terpene emission values, with the Tommy Atkins showing the highest average terpene signal intensity, ~870 ppbv, followed by the Keitt cultivar with ~750 ppbv. In contrast, the Sindhri cultivar recorded the lowest total terpene emission, ~13 ppbv, among the air-shipped mangoes. The Kensington Pride and Osteen cultivars, transported by truck from Italy and Spain, with terpene emissions of ~225 and 125 ppbv, respectively, showed intermediate terpene emission levels, closely aligning with those of the air-shipped mango from Israel (Figure 2). Similarly, the total emissions of ester compounds are clustered by transportation method. Ester emissions were highest in the air-shipped samples, with the Sindhri cultivar exhibiting the greatest signal intensity, followed by Kent from Peru and Mexico, respectively. The ester emission intensity in Sindhri was more than 20 times higher than that of the Tommy Atkins samples, which recorded the lowest ester emission among all varieties.

The PLS-DA approach was applied to identify VOCs that can discriminate among different shipping methods. By applying the PLS-DA on the mango fruit samples data, a correct distinction of the shipping methods was achieved independently of the mango cultivars. These results are consistent with previous studies [23,55,56], where VOC-based models successfully discriminated mangoes by ripening stage and different transport conditions. The optimal latent variable number associated with the minimum error rate (calibration and CV classification error average = 0.0000) and concurrently with the minimum number of not assigned samples (0.00) resulted in three LVs. The permutation test, evaluated using the Wilcoxon Signed Rank Test, R, and *t*-test, indicated that the model is significant at a 95% confidence level. The global quality of the model, evaluated by its performance indicators (Table 3), resulted in robustness to discriminate the mango samples in the model/validation data set and in the independent test set. Indeed, the PLS-DA three-component model successfully classified 100% of mango samples into their transport way category in fitting, cross-validation (internal validation), and prediction (external validation).

**Figure 3:**
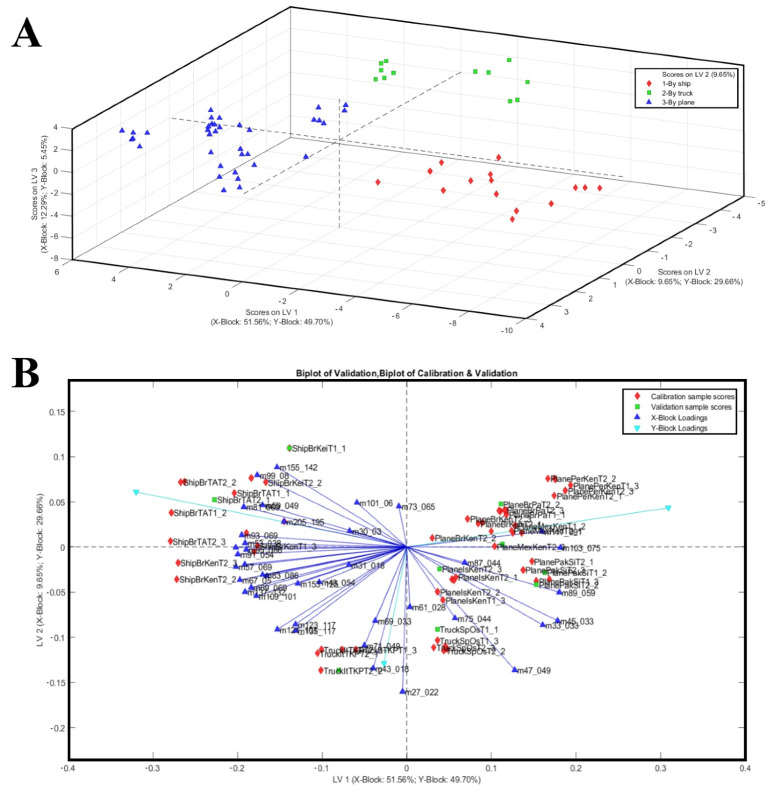
(**A**) The 3D score plot of the same PLS-DA model, illustrating class separation in three-component spaces. (**B**) PLS-DA biplot of mango samples based on VOC profiles, with transport methods (ship, truck, plane) used as Y-block classes. Samples are represented as black diamonds (ship), sky-blue squares (truck), and red triangles (plane).

Scores and a biplot of the PLS-DA model are shown in Figure 3, where the three-component PLS-DA model explained 73.49% of the X-block variance and 84.81% of the Y-block variance. The first latent variable (LV1) accounted for 51.56% of the X-block variance and 49.70% of the Y-block variance, providing a clear separation of the samples by transport mode. The 3D score plot (LV1 vs. LV2 vs. LV3) confirmed the complete discrimination of the groups in three-dimensional space. The PLS-DA model achieved a clear and robust classification of mango samples according to transport mode, revealing distinct VOC signatures that are intimately tied to the shipping methods and, therefore, the conditions imposed by different logistical chains (Figure 3A). Fruits transported by sea, which require early harvest to withstand prolonged shipping and cold storage, were characterised by volatile profiles dominated by terpene and C6 compounds (i.e., 81, 93, 99, 123, 135, 137, 153, 155, 205) typically associated with immature or less developed aromatic profiles (Figure 3A). Conversely, mangoes that underwent shorter and less invasive logistics, either air-shipped or truck-transported, showed a higher emission of alcohols, aldehydes, and esters, volatile classes closely linked with advanced ripening and heightened sensory appeal (i.e., *m*/*z* 33, 45, 47, 89, 103, 117). These findings align with the interpretation that transport mode, though indirect, exerts a profound influence on fruit quality by determining the maturity stage at which mangoes are harvested and how ripening proceeds thereafter. The PLS-DA model effectively discriminated mango samples according to transport mode based on VOC profiles. Indeed, the biplot highlighted the contribution of specific VOCs to group separation (Figure 3B).

In addition, to provide a more detailed characterization of the VOCs emitted by different shipping systems, only the *m*/*z* values presenting VIP scores higher than 1.2 and their possible identification are reported in Table 4. Particularly, as reported in Table 4, the compounds with highest VIP score for shipping discrimination were (**1**) for the boat-shipping system: *m*/*z* 81.069 (TI Terpene fragments), *m*/*z* 99.00 (TI 2-hexenal), *m*/*z* 137.132 (PI: acetaldehyde); (**2**) for the truck-shipping system: *m*/*z* 30.030 ethylene (isotope), *m*/*z* 47.049 (TI ethanol), *m*/*z* 101.060 (TI 3-Hexen-1-ol); (**3**) for the air-shipping system: *m*/*z* 45.033 (TI: acetaldehyde); *m*/*z* 95.086 (PI: C6 compounds/hexenol fragment) and *m*/*z* 99.080 (PI: 2-hexenal).

### 2.3. Sensory Evaluation and Consumer Acceptance by Trained and Untrained Panellists, Respectively

This section compares mango cultivars introduced to the Italian market via various transportation methods to identify the most effective shipping system for preserving quality and the key factors influencing consumer preference. Sensory evaluation with both trained and untrained panellists identified the sensory attributes most closely associated with consumer appreciation.

#### 2.3.1. Sensory Evaluation by Trained Panellists

**Figure 4:**
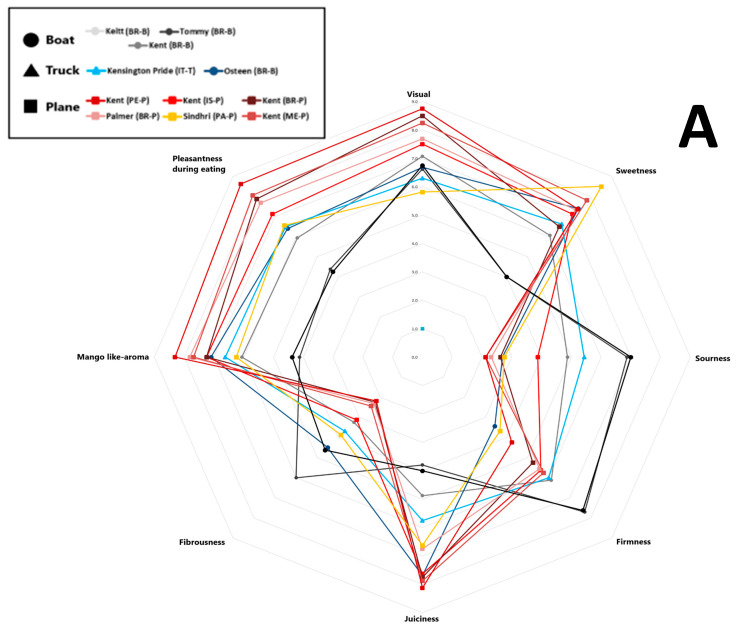

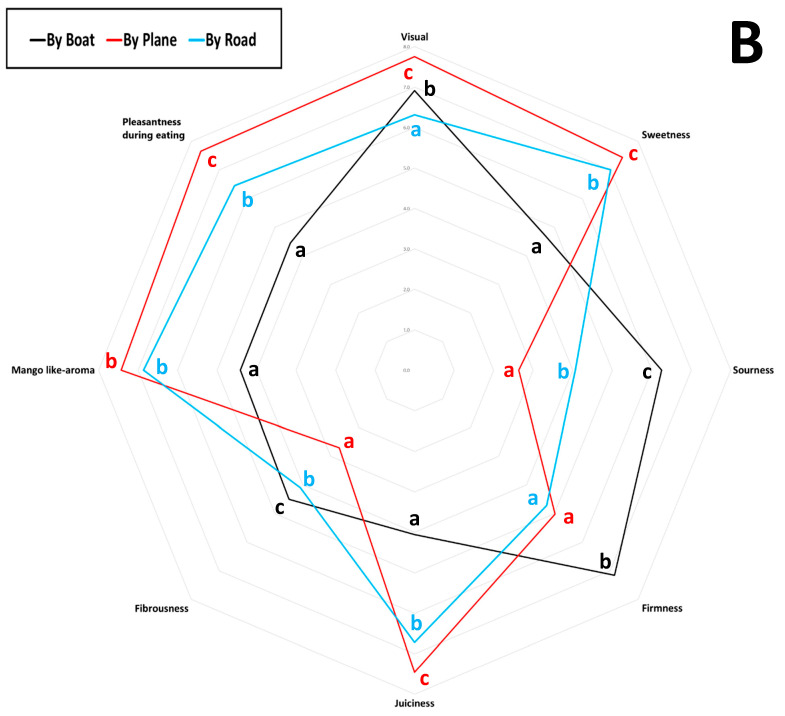
Sensory scores of eleven mango samples were evaluated over two consecutive years. Values are reported on a nine-point scale: 0–3 (very low to low), 3–4 (fair), 5–6 (medium), and 7–9 (high to highest). (**A**) Mango samples obtained from different cultivars, origins, and shipping methods; (**B**) samples grouped by different shipping methods. Different letters indicate differences by the LSD test at the 99.0% confidence level (*p* = 0.01).

The average scores for eight sensory attributes evaluated by panellists (visual appeal, sweetness, sourness, firmness, juiciness, fibrousness, mango-like aroma, and eating pleasantness) for each sample are represented in a spider plot, as shown in Figure 4. Specifically, Panel A presents the results for mango samples from different cultivars, origins, and shipping methods, while Panel B shows the samples grouped by shipping method. Trained panellist analysis revealed that the Kent mango cultivar air-shipped from Peru received the highest scores for visual appeal, juiciness, mango-like aroma, and overall pleasantness. It also earned the second-highest score for sweetness, following the Sindhri cultivar, which was rated the highest score. However, among the air-shipped mangoes, the Sindhri cultivar exhibited comparatively higher fibrousness, distinguishing it from the others. Overall, air-shipped mangoes displayed the lowest levels of fibrousness and sourness, with moderate firmness. Air-shipped Kent mangoes from Brazil, Mexico, and Peru were the most highly appreciated by panellists, based on their juiciness, sweetness, aroma, and pleasantness. In contrast, Kent mangoes from Israel were rated less favorably, perceived as sourer and more fibrous, with lower scores for visual appeal, sweetness, and overall pleasantness. Tommy Atkins, Keitt, and Kent cultivars transported by sea received the lowest ratings for sweetness, juiciness, aroma, and pleasantness, but scored the highest for sourness, firmness, and fibrousness. As noted by Sung et al. (2019) [53], mangoes are considered higher quality when they feature low fibrousness, intense sweetness, balanced acidity, and a fruity aroma profile. As shown in Figure 4, Panel B, mango samples arriving in Italy by air received the highest sensory scores for desirable attributes such as more pungent aroma, higher sweetness, greater juiciness, and enhanced eating pleasantness, clearly distinguishing them from fruit transported by sea or road. In contrast, sea-shipped mangoes scored higher for firmness, fibrousness, and sourness; traits often linked to lower maturity or suboptimal ripening conditions during transport [57]. Road-shipped samples displayed intermediate sensory scores, more like air-shipped than sea-shipped mangoes, particularly in terms of sweetness, aroma, and eating pleasantness.

#### 2.3.2. Consumer Acceptance

Mango quality was evaluated from the consumer’s perspective to identify the sensory attributes that most influence consumer acceptance. Mango quality was evaluated from the consumer’s perspective to identify the sensory attributes that most influence consumer acceptance. In this study, participants were asked to provide an overall rating after tasting each sample, without receiving any information about the fruit itself. Consumer acceptance was calculated for 11 different mango cultivars as the percentage of respondents who rated the sample 5 or higher on the hedonic scale. The results are presented in Table 5. Based on these evaluations, consumer acceptability scores were calculated for each mango sample.

**Figure 5:**
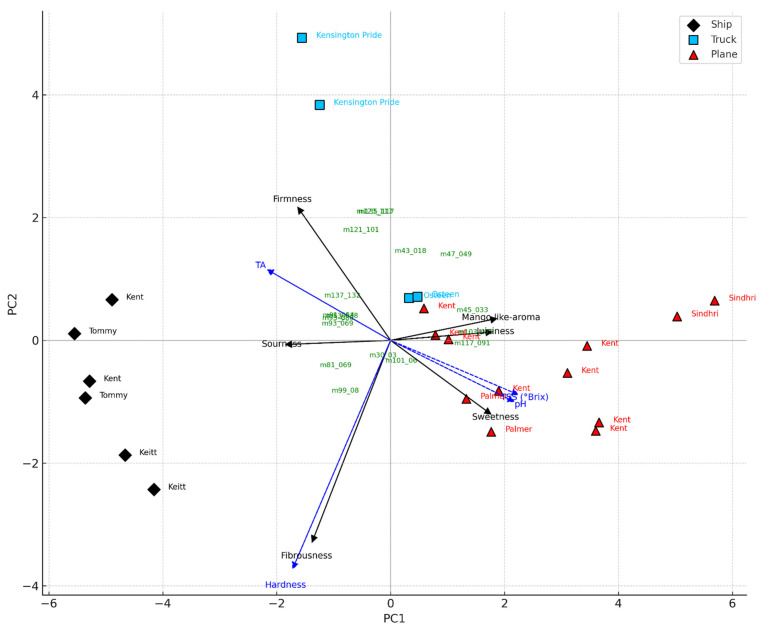
PCA triplot of mango samples transported by ship (black rhombus), truck (light blue square), and plane (red triangle), based on VOCs, physico-chemical, and sensory variables. Sensorial (black arrows) and physico-chemical (blue dashed arrows) variables are plotted as vectors. VOCs were plotted as green labels based on their projection on the principal components. Cultivar names were used as sample labels. The plot highlights the association between transport mode and fruit maturity traits.

Kent from Peru and Mexico, along with Palmer from Brazil, achieved the highest overall scores and consumer acceptability rates (all above 90%), while Tommy Atkins and Keitt received the lowest evaluations. Tommy Atkins from Brazil showed the poorest performance, with low scores in overall liking and acceptability (Table 4). Among the mangoes produced in Europe, the Kensington Pride cultivar from southern Italy showed an intermediate level of acceptability (72%), despite a relatively low overall rating (6.42 ± 0.60); in contrast, the Osteen cultivar from Spain was more highly appreciated. Moreover, significant differences (p** = 0.01) were observed based on the shipping method. Air-shipped samples received the highest average scores (7.61 ± 0.60), followed by those transported by road (6.62 ± 0.35), and finally those shipped by sea (5.33 ± 0.56). Among the air-shipped fruits, the Kent from Israel and the Sindhri cultivar had the lowest scores within this group. Road-transported samples showed intermediate consumer acceptability (over 70%), with the Osteen cultivar from Spain rated higher than the Italian Kensington Pride. Both road-shipped cultivars still outperformed the sea-shipped samples from Brazil. Sea-transported mango cultivars had the lowest consumer acceptability. Moreover, PCA applied to VOCs, physicochemical, and sensory variables revealed a clear separation of mango batches based on the transport method, highlighting the key attributes that drive consumer appreciation (Figure 5). The first two principal components accounted for 71.6% of the total variance (PC1: 58.6%, PC2: 13.0%), effectively distinguishing the samples by shipping mode. Mangoes transported by air were associated with higher sweetness, juiciness, and a mango-like aroma, consistent with elevated °Brix and pH values and a more favourable sensory profile. In contrast, sea-shipped samples were associated with higher firmness, greater titratable acidity, and lower consumer acceptance, likely due to earlier harvesting and suboptimal ripening resulting from prolonged transport times. Truck-transported mangoes displayed intermediate characteristics and were positioned between air- and sea-shipped samples in the PCA space. The loading vectors of sensory variables showed strong positive associations among sweetness, mango-like aroma, and juiciness on the positive side of PC1, while sourness and fibrousness were negatively associated, appearing on the negative axes of PC1 and PC2, respectively. Based on these results, the main quality attributes influencing consumer acceptability were sweetness, aroma, and juiciness, findings consistent with Liguori et al. (2018) [36], who identified flavour, aroma, sweetness, and juiciness as primary drivers of consumer liking. Similarly, the projection of VOCs revealed clustering patterns aligned with PLS-DA results. Esters and alcohols, typically associated with advanced ripening, clustered near the air-transported samples, while terpenes and C6 compounds, linked to green or under-ripe notes, were closer to sea-shipped mangoes (Figure 5). These observations align with previous findings on the influence of fruit maturity on sensory perception and consumer acceptance [58].

### 2.4. Comparative GHG Emissions and Emissions Intensity

The EcoTransIT analysis reveals significant variations in environmental impact across transport modalities, as reported in Table 6. Air freight demonstrates the highest CO_2_ emissions (CO_2_e), with exemplary cases being the Peruvian route (Piura-Florence) (0.7837 kg CO_2_e/t·km; 1,010,918 kg CO_2_e total) and the Mexican route (Guerreo-Mexico City-Florence) (0.6970 kg CO_2_e/t·km; 772,664 kg CO_2_e). The Punjab-Florence (0.6252 kg CO_2_e/t·km) and Haifa-Florence (0.7272 kg CO_2_e/t·km) routes exhibit comparable values, particularly in terms of emission intensity, which is attributed to geographical and operational factors, as demonstrated by Baumeister (2017) [59]. While aviation contributes only 0.4% to total food system emissions [60], its exceptional energy intensity (100–200 MJ/t·km) remains particularly critical, confirming the findings of Poore & Nemecek (2018) [61] regarding the imbalance between cargo volume and emission impact. Maritime transport, despite showing excellent energy efficiency (0.0185 kg CO_2_e/t·km on the Recife-Florence route), must account for additional trucking segments that contribute disproportionately (59.5% of total emissions while covering just 12.1% of the distance) and extend transit times to 18–25 days. This modal fragmentation significantly affects product quality, as demonstrated by Kailaku et al. (2023) [50]. Fruits subjected to maritime transport exhibit 30–40% reductions in soluble solids and 35% lower consumer acceptability due to the necessity of premature harvesting. In contrast, regional truck transport (Granada-Florence: 0,0913 kg CO_2_e/t·km; 17,585 kg CO_2_e total) represents the optimal balance between environmental sustainability and product quality. The shorter distances enable harvesting at more advanced maturity stages (≥85–90%), which preserves organoleptic characteristics while achieving significantly lower absolute emissions compared to air transport and generating fewer co-pollutants than air freight operations [62]. These combined advantages support the need to rethink supply models by prioritising local distribution channels, as suggested by recent literature [63], where distance reduction yields simultaneous environmental and quality benefits without requiring excessive compromises on either front.

## 3. Discussion

This study examined the availability and quality of mangoes in Tuscany, investigating how cultivar choice, shipping method, and environmental impact interact to influence the mangoes that ultimately reach consumers. The primary objective of this work was to document the mango cultivars available to consumers in the Florence metropolitan area (Tuscany region) during September of two consecutive years (2021–2022) and to evaluate how transportation practices may influence fruit quality and consumers’ choices. While the genetic background of each cultivar clearly plays a role, our results show that transportation is the single most decisive factor shaping both fruit quality and consumer appreciation.

Brazilian mangoes proved to be the most prevalent in Italian markets, as the third-largest global exporter [1], alongside South American samples, highlighting how this region remains one of the most significant exporters. Among the premium quality, only the Pakistani samples were shown to belong to the Indian group, while all the other samples belonged to the Indo-Caribbean/Floridian hybrids. This demonstrates a partial closure on the acceptability of different varieties of mangoes, emphasising the Kent varieties and similar varieties.

Sea-transported mangoes arrived firmer than air- or road-shipped fruit, a direct consequence of early harvesting and long cold-chain storage. Although firmness helps fruits withstand transit, it was also linked with higher acidity, lower sweetness, and reduced juiciness (Figure 5), ultimately lowering consumer acceptance. These patterns reflect the impact of prolonged storage, multiple handling steps, and delayed ripening during extended shipping [64]. By contrast, air and road shipments allowed harvesting at more advanced maturity, resulting in softer texture, lower acidity, and sweeter fruit profiles that consumers rated more positively [65,66]. Road-transported samples from European producers, such as Spanish Osteen and Italian Kensington Pride, fell between the extremes, demonstrating how local production paired with short logistics can deliver good quality while avoiding the drawbacks of maritime shipping.

Our results also confirm that transport methodologies strongly shape pomological parameters. Air-shipped mangoes consistently exhibited the lowest titratable acidity and the highest pH, TSS, and TSS/TA ratios, reflecting the natural degradation of organic acids and the proper accumulation of sugars. In contrast, sea-shipped mangoes showed the highest acidity, lowest TSS, and reduced TSS/TA ratios, consistent with incomplete ripening under cold storage [67]. Road-shipped mangoes displayed intermediate values, suggesting minimal stress and balanced flavour [68]. Notably, Sindhri mangoes achieved favourable TSS/TA ratios due to high sugar content, while Italian Kensington Pride maintained good quality levels despite lower sweetness compared to air-shipped cultivars. These examples illustrate how careful postharvest management [69] and local cultivation [70] can sustain consumer-acceptable quality with fewer environmental and economic costs. As previously mentioned, the storage conditions during transportation play a crucial role in maintaining the quality of the final product [25,71]. Although we did not evaluate different storage conditions in this study, it is essential to consider this aspect, given the significant percentage of waste produced during transportation resulting from improper storage conditions [24,71]. To preserve product quality, it is important to maintain shipping temperatures between 10–12.5 °C [24,71]. Additionally, utilising methods such as Modified Atmosphere Packaging and thermal insulation [25,28] can help retain firmness, limit weight loss, and prevent peel discolouration or internal damage. These measures can extend the product’s shelf life and reduce commercial rejection rates [24,71,72].

The sensory analysis and volatile profiling provided further insight into these differences. PCA results revealed that sweetness, juiciness, and aroma were the most influential quality attributes for sensory evaluation and consumer acceptance, consistent with previous findings [65,69]. These qualities were lowest in sea-transport samples, while sea-transported mangoes exhibited the highest fibrousness. This is particularly relevant as European consumers prefer fibre-free mangoes that are easy to slice [40]. Air-shipped mangoes stood out for their high levels of esters and ripening-related volatiles (Figure 5), compounds directly linked to sweetness, juiciness, and the characteristic mango-like aroma that consumers value most. By contrast, sea-shipped mangoes showed higher levels of terpenes and C6 compounds, typically associated with greener, less ripe flavours and fibrous textures. These findings were supported by multivariate analyses, which clearly separated transport modes according to their volatile signatures, confirming that logistics shape not only the physical traits of mangoes but also their aroma and flavour profile. To avoid influencing consumers’ decisions, panellists were not informed about costs, transportation systems, or country of origin, allowing for the evaluation of “ready-to-eat” mango preferences based exclusively on sensorial attributes. Previous studies demonstrate, for example, that pricing information significantly influences consumer choice, as price sensitivity effects often override quality perception [4,73,74]. Conversely, Spina et al. (2024) [75] reported that consumers’ purchasing decisions are increasingly less influenced by price, showing growing interest in mango organoleptic properties rather than economic factors. This trend toward quality-driven behaviour suggests promising market dynamics for premium mango products.

Thus, selecting the optimal shipping system involves balancing quality preservation, environmental impact, and economic considerations. Each transportation mode has a significant impact on both fruit quality and greenhouse gas emissions. Air freight demonstrates superior performance in quality maintenance, primarily due to the ability to harvest fruits at more mature, pre-ripened stages, which accommodates their short shelf life [22,35]. This method enables optimal flavour and aroma development, as demonstrated by our study. Moreover, air-transported samples consistently obtained the highest consumer appreciation rates, correlating with superior physicochemical parameters. However, the gas emissions per kilometre were the highest among the shipping methods tested, in line with what was reported by Padaliya and Pundir (2022) [76]. In contrast, sea transportation offers significant economic advantages and lower GHG emissions per kilometre [76]; however, this method substantially compromises final fruit quality, as demonstrated by pomological analyses and consumer acceptance results (Table 1 and Table 4). Indeed, the extended sea transport duration imposes a harvest at a mature-green stage to prevent fruit spoilage and damage during prolonged transit [77]. Therefore, promoting European mango production could favour truck transportation, which represents a promising compromise, offering GHG emissions per kilometre comparable to sea freight, while benefiting from much shorter distances and reduced transit times. The shorter supply chain enables harvesting closer to optimal maturity stages, enabling better preservation of desirable ripening characteristics [78]. Road-transported samples from European cultivation exhibited intermediate quality scores, indicating the potential for regional production to achieve acceptable quality with a reduced environmental impact. Therefore, our findings highlight a direct relationship between the transportation method and final product quality, indicating that the selection of the shipping system has a significant impact on market acceptance. Indeed, consumer appreciation is strongly influenced by organoleptic attributes and VOC profiles, which are directly affected by transportation methods.

## 4. Materials and Methods

### 4.1. Fruit Samples Collection

Mango fruit availability in the Tuscany region was assessed through surveys conducted in September 2021 and 2022, with a focus mainly on the Florence metropolitan area. Surveys were carried out at major large-scale retail outlets in the Florence area. During these surveys, mango fruits from six commercial cultivars (Kent, Keitt, Kensington Pride, Osteen, Tommy Atkins, and Palmer) were found and collected, as they were the cultivars available on the market during September. To increase varietal diversity, the Sindhri mango, a nearly unknown cultivar to the average Italian consumer, was also included, sourced from a local ethnic market. The collected mangoes were imported from seven different countries (Brazil, Israel, Italy, Mexico, Pakistan, Peru, and Spain) and transported via three different shipping methods: truck, air freight, and sea freight. Each sample was purchased at its retail price and subjected to analysis. In total, 11 distinct “ready-to-eat” mango samples were collected annually for each cultivar, for more than 250 individual fruits across the two years (23–25 fruits per each mango sample), to ensure consistency and representativeness. Upon acquisition, fruits were stored in a climate-controlled chamber at 10 °C and 85–90% relative humidity (RH) and analysed within 48 h of purchase. Before acquisition, all mangoes were visually inspected, and only uniform, defect-free fruits were selected for chemical-physical, volatile, and sensory analyses. No pathogens were detected in the selected samples, and no treatment was performed prior to the start of the analyses to prolong or enhance the preservation of the samples. A detailed list of all samples included in the study is provided in Figure 1, which shows mango varieties along with their transport modes, origins, price ranges, morphological types, and the related greenhouse gas (GHG) emissions associated with each transport mode per kilometre travelled.

### 4.2. Analysis of Pomological Character (Total Soluble Solids (TSSs), Titratable Acidity (TA), pH, Flesh Firmness (FF), and Ratio (TSS/TA)

Mango fruits were washed, drained, and gently dried with paper towels. Pomological measurements were performed at the equatorial region of each fruit and were based on the average of five fruits per sample (5 fruits per cultivar repeated for each sampling year). Fruit weight was immediately recorded using a precision balance with an accuracy of 0.01 g. To assess fruit firmness, a digital penetrometer (T.R. Turoni S.r.l., 53205, Forlì, Italy) was equipped with a round tip (8 mm diameter). Data were expressed as the force (kg cm^−2^) required to rupture the pulp, after the peeling of the area through a blade. Subsequently, the pulp was manually separated from the fruit and cut into small pieces to ensure sample homogeneity. pH, total soluble solids (TSSs), and titratable acidity (TA) were then assessed. To determine the pH, 15 g of small pieces of fruit were shredded and homogenised with 150 mL of deionised water. The resulting mixture was filtered, and the pH was measured at room temperature using a pH meter, Basic 20 Crison. Titratable acidity expressed as g citric acid/L was evaluated using a CrisonS compact titrator (Crison Instruments, SA, Barcelona, Spain). Finally, total soluble solids (TSSs) were determined with a digital refractometer (Atago Co., Ltd.,Fukuya City, Saitama, Japan). Moreover, the TSS/TA ratio, a dimensionless parameter derived by dividing total soluble solids (TSSs) by titratable acidity (TA), was calculated to evaluate the balance between sweetness and acidity in each mango sample [79].

### 4.3. PTR-ToF-MS Acquisition

The PTR-TOF 8000 (Ionicon Analytik GmbH, Innsbruck, Austria) is a high-sensitivity, high-resolution mass spectrometer [80]. In this study, H_3_O^+^ has been used as a reagent ion. The operating principle of the PTR–ToF–MS technique is described in detail elsewhere [81]. Instrument setup and sample preparation followed the protocol established by Taiti et al. (2016) [23] for mango fruits. Specifically, ~5 g of pulp cubes was collected from three different fruits for each mango cultivar and used to obtain at least three replicates (*n* = 11 cultivars × 2 years × 3 replicates). Specifically, for each acquisition, fresh mango pulp (excluding the peel) was placed in a 250 mL glass jar and incubated for 60 s. Before measurement, the jar’s headspace was purged with clean (zero) air to eliminate residual VOCs. During analysis, purified air continuously flowed through the jar, carrying headspace volatiles into the PTR inlet. Once the sample was analysed, it was then cleaned in the ToF inlet. All measurements were performed in a temperature and humidity-controlled chamber (25 °C, constant relative humidity) to minimise variability in VOC partitioning. The drift tube was operated at a temperature of 60 °C and a pressure of 2.2 mbar, corresponding to an electric field-to-neutral gas number density ratio (E/N) of 130 Td (1 Td = 10^−17^ cm^2^ V^−1^ s^−1^). The sampling time per channel in the time-of-flight (ToF) analyser was set to 0.1 ns. For each sample, data acquisition lasted at least 80 s, and the resulting spectra were averaged over this interval to generate a representative VOC profile. Internal calibration was based on *m*/*z*  =  29.997 (NO^+^), *m*/*z*  =  59.049 (C_3_H_7_O^+^) and *m*/*z*  =  137.132 (C_10_H_17_^+^) and was performed off-line. Blank measurements of empty jars under identical conditions were also recorded and subtracted as background. Raw spectra (*m*/*z* 20–220) were processed to extract signals corresponding to target compounds. Acquisition of raw data and quantification of the peaks were performed according to a procedure described by Cappellin et al. (2011) [82]. Compound identification was based on their exact *m*/*z* values and characteristic PTR-MS fragmentation patterns, as referenced in published spectra [56,83,84]. To ensure data reliability and minimise noise from trace-level signals, a threshold of 1 ppbv was applied. This threshold was calculated as the average concentration across five replicates. Compounds with an average concentration below one ppbv were undetectable and were therefore excluded from the final dataset.

### 4.4. Sensory Measurements by Trained and Untrained Panellists

#### 4.4.1. Sample Preparation

Mango samples were used for both trained panel and consumer sensory evaluations. Each fruit was cut into 2 cm^3^ pulp cubes (peel excluded) and served in separate evaluation sessions. For each sample, at least three peeled samples were presented to each panellist within one hour of cutting to minimise browning. Samples were coded with randomly assigned three-digit numbers, and the order of presentation was randomised for each panellist. Mineral water was provided to participants for palate cleansing between samples. After the visual determination (whole fruit), each judge could taste the mango to assess it twice, if needed. To avoid bias from intrinsic or extrinsic cues (e.g., price, origin, ripeness), no such information was provided [85].

#### 4.4.2. Panel Test by Trained Panellists

Sensory profile analysis was conducted according to the UNI 10957 (2003) [86] by a trained panel of eight judges (aged 30–55), each qualified in the sensory evaluation of various fruit types. The sensory evaluation, conducted by trained panellists, was repeated twice over two consecutive years (2021 and 2022). In preliminary sessions, the panel selected ten sensory descriptors: visual appearance, sweetness, sourness, firmness, juiciness, fibrousness, mango-like aroma, and overall pleasantness during eating. Evaluations were performed in individual booths under controlled lighting and temperature conditions. Each judge assessed the samples in duplicate, using two different fruits from each cultivar per session. The sample order was randomised for each panellist, and water was provided for palate cleansing between samples. Judges scored each descriptor using a discontinuous 9-point intensity scale, where 1 indicated the absence of the sensation and 9 indicated maximum perceived intensity [37].

#### 4.4.3. Consumer Acceptability by Untrained Panellists

A one-year trial was conducted involving a consumer evaluation with 65 untrained Italian participants (aged 19–65), recruited from the University of Florence. While familiar with mangoes, they had no prior experience with sensory evaluation. Participants assessed differences and acceptability of mango samples using a nine-point hedonic scale (1 = dislike extremely, 9 = like extremely) [87]. A score of six marked the threshold for commercial acceptability; consumer acceptability was defined as the percentage of scores above 5 [88,89].

### 4.5. Greenhouse Gas Evaluation

This analysis was performed using EcoTransIT World 2024, a web-based ecological transport emissions calculator developed by the Institute for Energy and Environmental Research (ifeu), certified under the GLEC Framework v3.1 [89,90]. Our study quantifies greenhouse gas (GHG) emissions associated with the international transportation of mangoes to Europe, adopting a multimodal, door-to-door approach consistent with internationally recognised methodologies. According to the literature, each transport route begins in the central production region of the exporting country and ends in Florence, Italy, which is chosen as a representative inland logistics hub. According to Müller-Carneiro et al. (2023) [13], Brazilian mango production is primarily concentrated in the Vale do São Francisco, near Recife in Pernambuco. For Peruvian mangoes, the Piura region was used as a reference [91]. In Mexico, production is mainly located in the state of Guerrero [92]. In Pakistan, the world’s fourth-largest exporter, the Punjab region represents the principal production area [93]. In Israel, cultivation is mainly concentrated in the northern part of the country, near Haifa [94]. Within Europe, Sicily is regarded as the primary production region in Italy, whereas in Spain, Andalusia is the leading production area [95]. GHG emissions have been taken into consideration, given the transition to the Netherlands, a country with a central distribution centre for imported products, the Rotterdam port for sea-shipping products, and Amsterdam Schiphol Airport for air-freighted products [96]. GHG emissions were calculated for a reference shipment of one metric ton (gross weight) of tropical fruit. For maritime shipments, emissions were scaled based on an average container load of 10 metric tons per TEU (Twenty-Foot Equivalent Unit). Intermodal transport chains (e.g., air and truck, or sea and truck) systematically include both pre-carriage and on-carriage legs. For instance, the Recife-Florence maritime route in Brazil includes 1319 km of pre-haulage by truck, followed by 8939 km of sea transport to Rotterdam port, and a final road segment of 1312 km to Florence. EcoTransIT World computed all distances based on actual operational routes, including technical stopovers and realistic detours, rather than idealised straight-line paths. Operational parameters were defined using average real-world values. A 70% load factor was applied to air freight (Boeing 747-400F freighter), and 55% for maritime vessels (general cargo, 35,000–80,000 DWT). For road transport (Euro 6 diesel trucks, Class 26), an empty trip factor of 20% was incorporated to account for partial backhauls. The emissions calculation follows a Well-to-Wheel (WTW) approach, incorporating both Well-to-Tank (WTT) emissions from fuel production and distribution, as well as Tank-to-Wheel (TTW) emissions from the combustion process. Greenhouse gases (CO_2_, CH_4_, N_2_O) are converted into CO_2_ equivalents (CO_2_e) using GWP factors from the IPCC AR6 [97]. The resulting emissions are then normalised to kg CO_2_e per ton-kilometre by dividing total WTW emissions by the product of total door-to-door distance and transported mass. Results were validated against DEFRA benchmarks (DEFRA, 2023) for air (0.48–0.57 kg CO_2_e/t-km) and road freight (0.09–0.11 kg CO_2_e/t-km), and IMO values for maritime shipping (typically 0.01–0.025 kg CO_2_e/t-km). Minor deviations (generally ±2%) reflect operational variability such as weather disruptions or routing constraints. To ensure robustness, the analysis includes correction factors for partial load utilisation and considers regulated air pollutants (NO_x_, SO_2_, PM) alongside direct GHGs. This integrative method provides a transparent and reproducible framework for assessing the climate impact of global perishable supply chains, supporting data-driven sustainability evaluations.

### 4.6. Statistical Data Analysis

One-way analyses of variance (one-way ANOVA) were performed to compare the physicochemical data (Weight, Firmness, TSS-°Brix, TA, pH, TSS/TA ratio), the sensory evaluation, and the consumer acceptability results, respectively; the separation of means was calculated by Fisher’s least significant difference (LSD) test. Computations were performed by Statgraphics Centurion XV v. 15.0.04.

#### 4.6.1. PLS-DA Analysis on Mango Samples by Transport Class

Partial Least Squares Discriminant Analysis (PLS-DA) (supervised method) was performed to discriminate mango samples according to their transport mode (ship, truck, plane) based on their volatile organic compounds (VOCs) profile obtained by Proton Transfer Reaction–Time of Flight–Mass Spectrometry (PTR-ToF-MS). The dataset comprised 66 mango samples (11 cultivars × 2 years × 3 replicates) from different cultivars and origins, with VOC profiles consisting of 41 selected masses. As a pre-processing step, data were submitted to logarithmic transformation (log_10_ + 1). Both X-block (VOCs) and Y-block (transport class) were autoscaled prior to analysis. The PLS-DA model was developed using PLS_Toolbox (Eigenvector Research Inc., version 9.2) implemented in MATLAB R2017b. The dataset was divided into a training set (85% of the samples) and a test set (15%) using the Kennard and Stone algorithm (1969) [98], which ensures the selection of a representative sample based on Euclidean distances. The training set was used to select the optimal number of latent variables, calibrate the model, and perform internal validation through venetian blind cross-validation. The model’s ability to correctly classify samples was then evaluated on the independent test set. Model performance was assessed using cross-validation and prediction errors (RMSECV and RMSEP) as well as confusion matrices. The number of latent variables was optimised by minimising both calibration error and misclassification rates. The classification threshold was chosen to minimise false positives and negatives, following Bayes’ theorem. Additionally, Variable Importance in Projection (VIP) scores (*p* = 0.01) were used to identify the VOCs that contributed most significantly to group separation. To help identify possible overfitting of the model, a permutation test (60 iterations) was also conducted, generating nonsense datasets for comparison with the real model. This procedure provides a probability that the given model is significantly different from one built under the same conditions but on random data.

#### 4.6.2. PCA Analysis on Mango Samples from Different Origins and Transport Systems

A Principal Component Analysis (PCA) was performed to investigate the relationship between sensorial attributes, physico-chemical parameters, and volatile organic compounds (VOCs) in mango samples transported via three different systems: ship, truck, and plane. The dataset included average values for several mango batches differing in cultivar and transport method (ship, truck, plane). In particular, the following variables were considered: (1) sensorial variables: sweetness, sourness, firmness, juiciness, fibrousness, mango-like aroma, evaluated by a trained panel; (2) physico-chemical variables: pH, titratable acidity (TA), soluble solids content (°Brix), and hardness (firmness measured by penetrometer); (3) VOCs: 17 protonated mass peaks selected as VIPs from a prior PLS-DA (log10 + 1 transformed). The PCA was performed on centred and scaled X-block data (sensorial, physico-chemical, and VOCs). The transport mode was used for visual encoding but not for dimensionality reduction. PCA was computed using Python 3.11.

## 5. Conclusions

This study reveals that the mode of transportation acts as a key factor influencing all quality attributes. It suggests that optimising the supply chain requires an integrated approach that simultaneously considers quality, environmental impact, and economic factors. The high quality associated with air transport may justify a premium pricing strategy in markets where consumers prioritise quality over price. Meanwhile, developing regional production in Europe could provide sustainable alternatives with lower environmental impact while maintaining acceptable quality levels. The findings advocate for the creation of transportation-specific quality standards and marketing strategies, acknowledging that different transportation modes deliver distinct products to consumers. Future research should concentrate on optimising harvest timing and postharvest treatments tailored to each transportation mode. It should also focus on developing cultivars better suited for extended transport and exploring consumer willingness to pay for quality in relation to environmental concerns. Furthermore, integrating advanced packaging technologies can enhance cold chain management and precision in harvest timing across all modes of transportation. This represents a promising opportunity for improving the sustainability and quality of mango supply chains targeting European markets.

## Figures and Tables

**Table 1:** Comparison of pomological characteristics among different mango cultivars, countries of origin, and transportation methods. The values represent the mean measurements for each sample collected over two consecutive years (*n* = 5 × 2 years), accompanied by the Standard Deviation (SD). Different capital letters indicate differences by the LSD test at the 99.0% confidence level (*p* = 0.01). * Descriptor list for Mango [48].

Mango Cultivar	Mango Fruit and Shape Descriptor *	Transportation Mode	Fruit Origin	Weight (g)	Firmness (kg/cm^2^)	TSS (°Brix)	TA	pH	TSS/TA Ratio
Tommy Atkins	3 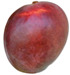	Boat	Brazil	557 ± 33	2.73 ± 0.12	12.50 ± 0.5	4.63 ± 0.23	3.54 ± 0.42	2.70 ± 0.29
Kent	3 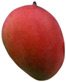	Boat	Brazil	475 ± 86	2.32 ± 0.25	13.00 ± 0.5	4.05 ± 0.20	3.73 ± 0.23	3.21 ± 0.25
Keitt	4 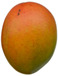	Boat	Brazil	502 ± 19	2.36 ± 0.40	12.55 ± 0.5	4.20 ± 0.43	3.67 ± 0.24	3.00 ± 0.40
Osteen	1 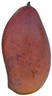	Truck	Spain	548 ± 19	1.16 ± 0.16	16.00 ± 0.7	3.00 ± 0.30	4.10 ± 0.34	5.35 ± 0.50
Kensington Pride	3 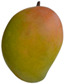	Truck	Italy	419 ± 24	1.25 ± 0.35	14.50 ± 0.5	3.35 ± 0.25	4.05 ± 0.40	4.33 ± 0.25
Kent	3 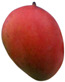	Plane	Israel	461 ± 26	1.18 ± 0.15	14.50 ± 0.4	3.10 ± 0.35	4.12 ± 0.17	4.70 ± 0.35
Kent	3 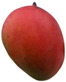	Plane	Brazil	434 ± 27	1.09 ± 0.20	15.80 ± 0.6	3.12 ± 0.21	4.10 ± 0.38	5.02 ± 0.20
Palmer	1 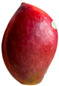	Plane	Brazil	533 ± 14	1.05 ± 0.14	16.20 ± 0.5	2.90 ± 0.25	4.03 ± 0.20	5.60 ± 0.31
Kent	3 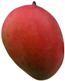	Plane	Mexico	578 ± 31	1.28 ± 0.25	16.75 ± 0.8	1.60 ± 0.54	4.43 ± 0.12	10.47 ± 1.4
Kent	3 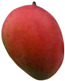	Plane	Peru	524 ± 13	0.93 ± 0.16	16.50 ± 0.9	2.25 ± 0.31	4.20 ± 0.28	7.33 ± 0.95
Sindhri	2 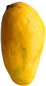	Plane	Pakistan	282 ± 23	0.74 ± 0.18	18.75 ± 1.3	1.46 ± 0.50	4.73 ± 0.25	12.80 ± 1.9
By Boat				511 ± 42 A	2.47 ±0.22 B	12.7 ± 0.3 A	4.29 ± 0.43 B	3.69 ± 0.29 A	2.97 ± 0.25 A
By Plane				469 ± 105 A	1.03 ± 0.18 A	16.4 ± 1.4 B	2.41 ± 0.81 A	4.39 ± 0.33 B	7.65 ± 3.30 C
By Truck				484 ± 70 A	1.16 ± 0.21 A	15.3 ± 1.1 B	3.42 ± 0.35 A	4.11 ± 0.14 B	4.83 ± 0.71 B

**Table 2:** Number of detected volatile organic compounds (VOCs), mass-to-charge (*m*/*z*) ratios, tentative identifications (TIs), chemical formulas, and average concentrations ± standard deviation, calculated over two years (2021–2022) of trials for each mango sample analysed. At the bottom of the table, total VOC emission values are reported, including total VOC emission (calculated as the sum of all detected VOCs), total terpene emission (calculated as the sum of *m*/*z* 67, 81, 91, 93, 95, 109, 121, 123, 135, 137), total ester emission (calculated as the sum of *m*/*z* 89, 103, 117), and ripening-associated VOC emission (calculated as the sum of *m*/*z* 33, 45, 47).

Sample ID				1	2	3	4	5	6	7	8	9	10	11
Sample Origin				Brazil	Brazil	Brazil	Spain	Italy	Israel	Brazil	Brazil	Mexico	Peru	Pakistan
Transportation System				Ship	Ship	Ship	Truck	Truck	Plane	Plane	Plane	Plane	Plane	Plane
**Cultivar**				Tommy Atkins	Keitt	Kent	Osteen	Kensington Pride	Kent	Kent	Palmer	Kent	Kent	Sindhri
**N°**	*m*/*z*	Chemical Formula	Tentative Identification	Average + SD	Average + SD	Average + SD	Average + SD	Average + SD	Average + SD	Average + SD	Average + SD	Average + SD	Average + SD	Average + SD
1	27.022	C_2_H_3_^+^	Acetylene	195.09 ± 49.6	103.5 ± 21.4	376.5 ± 21.4	1466.7 ± 472.2	313.4 ± 111.9	569.1 ± 101.4	155.3 ± 53.0	154.2 ± 46.4	187.3 ± 38.6	148.9 ± 30.6	250.6 ± 104.8
2	30.030	C_2_H_6_^+^	Ethylene (isotope)	19.2 ± 5.3	9.6 ± 3.1	19.7 ± 8.5	9.8 ± 5.0	5.3 ± 5.1	13.4 ± 3.9	17.0 ± 5.7	8.3 ± 4.9	6.6 ± 3.2	4.2 ± 1.4	19.0 ± 7.2
3	31.018	CH_3_O^+^	Formaldehyde	182.2 ± 56.6	6.2 ± 2.5	32.8 ± 12.2	10.1 ± 2.9	34.6 ± 6.8	30.3 ± 8.2	30.3 ± 6.7	7.6 ± 2.5	12.6 ± 2.8	20.8 ± 9.2	75.2 ± 5.3
4	33.033	CH_5_O^+^	Methanol	197.2 ± 50.4	278.6 ± 106.8	397.6 ± 96.6	2509.1 ± 778.3	544.1 ± 84.0	1059.5 ± 335.7	1178.7 ± 293.0	1287.6 ± 301.0	931.7 ± 411.1	941.8 ± 229.4	2918.1 ± 446.1
5	41.038	C_3_H_5_^+^	Fragments	166.3 ± 74.3	113.6 ± 25.8	71.9 ± 25.1	37.0 ± 5.1	61.0 ± 14.5	26.8 ± 9.	18.0 ± 6.0	16.8 ± 2.1	29.5 ± 9.7	6.9 ± 2.8	80.3 ± 8.5
6	43.018	C_2_H_3_O^+^	Acetates fragment	225.8 ± 78.1	22.6 ± 5.1	191.7 ± 72.8	618.4 ± 324.4	125.4 ± 20.4	97.3 ± 12.0	166.8 ± 51.2	23.2 ± 2.7	90.5 ± 33.5	13.8 ± 7.0	619.9 ± 55.0
7	43.054	C_3_H_7_^+^	Fragments (e.g., propanal)	90.9 ± 30.6	45.5 ± 9.5	52.5 ± 31.1	24.4 ± 3.5	53.7 ± 12.1	14.9 ± 4.7	22.6 ± 9.5	8.8 ± 1.1	17.4 ± 6.3	13.9 ± 5.4	244.3 ± 44.5
8	45.033	C_2_H_5_O^+^	Acetaldehyde	460.8 ± 103.4	477.4 ± 25.2	556.4 ± 52.2	2273.0 ± 592.1	1488.7 ± 409.9	1732.9 ± 348.4	1539.2 ± 354.2	1769.7 ± 371.9	2256.1 ± 482.2	1867.4 ± 533.5	2916.1 ± 469.3
9	47.049	C_2_H_7_O^+^	Ethanol	15.0 ± 3.2	15.9 ± 3.7	22.9 ± 2.9	160.9 ± 27.4	65.4 ± 26.8	60.7 ±13.8	61.5 ±15.2	31.0 ± 3.6	49.0 ±14.9	46.0 ± 14.4	215.2 ± 44.5
10	53.038	C_4_H_5_^+^	Fragments	40.6 ± 19.1	25.7 ± 5.1	17.6 ± 5.7	7.2 ± 0.9	12.6 ±4.5	2.4 ± 1.6	1.9 ± 0.7	6.1 ± 2.8	4.5 ± 1.4	1.3 ± 0.7	0.7 ± 0.1
11	55.054	C_4_H_7_^+^	Butadiene/Alkyl Fragments	42.9 ± 18.6	23.6 ± 5.0	27.9 ±25.2	7.4 ± 1.1	25.0 ± 3.1	6.6 ± 1.8	8.1 ± 2.7	3.5 ± 0.4	4.1 ± 1.5	4.3 ± 1.8	4.7 ± 0.6
12	57.033	C_3_H_5_O^+^	Fragments (e.g., Butanol fragment)	42.9 ± 18.6	23.6 ± 5.0	27.9 ± 25.2	7.4 ± 1.1	25.0 ± 3.1	6.6 ± 1.8	8.1 ± 2.7	3.5 ± 0.4	4.1 ± 1.5	4.3 ± 1.8	4.7 ± 0.6
13	57.069	C_4_H_9_^+^	Alkyl fragment	31.4 ± 13.2	18.9 ± 3.9	29.3 ± 14.6	6.3 ± 0.8	12.4 ± 4.2	6.9 ± 0.6	3.0 ± 1.4	2.8 ± 0.3	3.6 ± 1.2	1.0 ± 0.4	1.2 ± 0.3
14	59.049	C_3_H_7_O^+^	Propanal, Acetone	219.3 ± 126.9	114.8 ± 2.4	220.3 ± 83.4	32.3 ± 8.7	53.8 ± 5.3	27.8 ± 4.1	42.6 ± 17.0	22.6 ± 6.4	32.4 ± 13.8	61.1 ± 31.7	20.3 ± 2.4
15	61.028	C_2_H_5_O_2_^+^	Acetates	48.8 ± 10.3	17.9 ± 5.1	71.5 ± 25.5	83.4 ± 33.0	35.6 ± 13.7	24.8 ± 3.8	53.6 ± 16.3	25.1 ± 7.6	22.2 ± 5.3	28.1 ± 8.8	337.7 ± 75.0
16	67.054	C_5_H_7_^+^	Terpene fragments	31.4 ± 13.7	15.3 ± 3.1	31.7 ± 10.9	5.5 ± 0.7	17.8 ± 6.2	4.3± 0.4	3.9 ± 1.5	2.8 ± 0.4	3.0 ± 1.0	0.0 ± 0.0	1.1 ± 0.8
17	69.033	C_4_H_5_O^+^	Furan	8.3 ± 3.5	4.4 ± 0.8	8.7 ± 5.6	17.5 ± 3.6	5.2 ± 1.8	1.9 ± 0.2	1.8 ± 0.6	1.6 ± 0.2	0.9 ± 0.3	1.4 ± 0.4	162.4 ± 21.5
18	69.069	C_5_H_9_^+^	Isoprene	41.8 ± 17.4	24.3 ± 5.4	33.9 ± 17.5	8.2 ± 2.3	22.8 ± 3.2	6.7 ± 0.6	3.5 ± 1.2	4.5 ± 0.6	5.0 ± 1.7	0.9 ± 0.3	14.9 ± 2.0
19	71.049	C_4_H_7_O^+^	Ethyl butanoate	2.1 ± 0.9	1.1 ± 0.6	15.7 ± 18.1	6.5 ± 2.4	2.4 ± 0.9	1.7 ± 0.1	1.7 ± 0.5	0.3 ± 0.3	0.9 ± 0.3	0.0 ± 0.0	25.9 ± 2.4
20	73.065	C_4_H_9_O^+^	Methyl ethyl ketone	1.7 ± 0.6	1.0 ± 0.4	6.9 ± 7.3	0.9 ± 0.3	0.8 ±0.2	0.8 ± 0.1	5.3 ± 2.1	1.9 ± 0.8	0.0 ± 0.0	3.6 ± 1.6	1.8 ± 0.2
21	75.044	C_3_H_7_O_2_^+^	Butanol/Methyl acetate	15.2 ± 8.1	24.8 ± 11.5	43.7 ± 35.8	31.6 ± 6.7	54.8 ± 32.5	34.8 ± 10.6	87.2 ± 23.4	26.6 ± 4.8	66.3 ± 32.1	10.3 ± 4.6	225.2 ± 34.5
22	77.038	C_6_H_5_^+^	Alkyl fragment	20.4 ± 6.6	11.8 ± 2.5	14.6 ± 8.1	2.7 ± 0.3	7.4 ± 2.1	2.6 ± 0.2	3.2 ± 1.6	1.8 ± 0.2	1.9 ± 0.6	0.6 ± 0.3	0.7 ± 0.1
23	81.069	C_6_H_9_^+^	Terpene fragments	604.9 ± 94.9	543.4 ± 80.1	444.8 ± 49.2	59.6 ± 19.5	53.5 ± 16.9	51.0 ± 11.5	39.6 ± 17.1	35.1 ± 8.6	24.8 ± 8.1	17.6 ± 5.4	6.7 ± 2.5
24	83.086	C_6_H_11_^+^	C6 compounds/hexenol fragment	5.3 ± 3.6	1.0 ± 0.4	13.4 ± 8.0	0.9 ± 0.8	3.3 ± 0.4	0.7 ± 0.0	1.9 ± 0.7	0.6 ± 0.4	0.4 ± 0.1	0.0 ± 0.0	0.3 ± 0.1
25	87.044	C_4_H_7_O_2_^+^	2-Butenoic acid	2.5 ± 1.4	1.1 ± 0.6	14.7 ± 3.6	10.2 ± 3.8	1.6 ± 0.6	1.9 ± 0.5	3.9 ± 1.5	3.2 ± 1.9	6.9 ± 3.2	13.1 ± 5.3	88.6 ± 11.2
26	89.059	C_4_H_9_O_2_^+^	Ethyl acetate	12.1 ± 4.4	17.1 ± 7.0	46.6 ± 19.9	105.3 ± 31.9	59.7 ± 19.8	76.4 ± 26.7	104.1 ±38.8	95.3 ± 30.0	158.0 ± 63.9	171.0 ± 66.1	348.0 ± 103.5
27	91.054	C_7_H_7_^+^	Monoterpene fragments	8.5 ± 2.1	5.3 ± 1.1	16.3 ± 4.6	1.2 ± 0.2	3.9 ± 1.4	1.4 ± 0.0	1.0 ± 0.7	1.1 ± 0.8	0.8 ± 0.3	0.0 ± 0.0	0.8 ± 0.8
28	93.069	C_7_H_9_^+^	Terpene fragments	36.1 ± 6.3	16.3 ± 1.3	58.8 ± 21.2	3.5 ± 0.9	10.7 ± 3.5	1.6 0.7	5.5 ± 2.1	1.2 ± 0.4	1.0 ± 0.3	0.9 ± 0.3	0.0 ± 0.0
29	95.086	C_7_H_11_^+^	Terpene fragments	123.8 ± 41.9	120.1 ± 32.3	82.4 ± 13.2	30.3 ± 5.9	71.6 ± 19.7	17.1 ± 4.6	9.7 ± 4.6	14.0 ± 1.8	10.9 ± 3.4	9.7 ± 4.8	1.7 ± 0.2
30	99.080	C_6_H_11_O^+^	(E)-2-hexenal	6.6 ± 2.5	5.9 ± 2.0	5.6 ± 3.1	0.5 ± 0.3	0.7 ± 0.1	0.6 ± 0.3	0.7 ± 0.1	0.6 ± 0.3	0.7 ± 0.1	0.6 ± 0.3	0.7 ± 0.1
31	101.060	C_6_H_13_O^+^	3-Hexen-1-ol	2.4 ± 1.0	2.4 ± 0.5	1.3 ± 0.7	0.3 ± 0.0	0.3 ± 0.1	0.5 ± 0.0	0.5 ± 0.0	0.5 ± 0.0	0.5 ± 0.0	0.4 ± 0.1	7.2 ± 3.1
32	103.075	C_5_H_11_O_2_^+^	Methylbutanoic acid/Methyl butanoate	4.9 ± 1.9	4.1 ± 0.5	5.1 ± 2.5	11.2 ± 3.7	23.6 ± 7.0	30.4 ± 9.0	34.4 ± 11.8	38.7 ± 12.4	36.6 ± 15.9	92.2 ± 37.0	71.3 ± 8.3
33	109.101	C_8_H_13_^+^	Terpene fragments	7.2 ± 3.3	1.9 ± 0.4	8.1 ± 4.7	0.9 ± 0.3	6.2 ± 3.2	4.7 ± 3.7	0.3 ± 0.2	0.7 ± 0.5	0.9 ± 0.1	0.0 ± 0.0	0.0 ± 0.0
34	117.091	C_6_H_13_O_2_^+^	Ethyl butanoate/Isobutyl acetate	4.6 ± 1.6	2.7 ± 0.1	5.0 ± 0.7	8.9 ± 4.1	7.8 ± 1.4	6.5 ± 2.6	12.5 ± 4.2	15.7 ± 3.1	37.6 ± 19.7	60.7 ± 21.2	76.5 ± 6.5
35	121.101	C_9_H_13_^+^	Monoterpene fragments	2.3 ± 0.5	1.0 ± 0.6	3.9 ± 3.5	0.7 ± 0.3	7.3 ± 2.5	2.4 ± 1.2	0.2 ± 0.1	0.1 ± 0.3	0.0 ± 0.0	0.0 ± 0.0	0.0 ± 0.0
36	123.117	C_9_H_15_^+^	Terpene fragments	1.1 ± 0.8	0.0 ± 0.0	1.5 ± 0.6	0.0 ± 0.0	1.8 ± 0.6	0.0 ± 0.0	0.0 ± 0.0	0.0 ± 0.0	0.0 ± 0.0	0.0 ± 0.0	0.0 ± 0.0
37	135.117	C_10_H_15_^+^	Terpene, e.g., p-Cymene/a-Cedrene	1.3 ± 0.5	0.5 ± 0.1	7.3 ± 4.9	0.0 ± 0.0	12.6 ± 5.2	1.5	0.4	0.0 ± 0.0	0.0	0.0	0.0 ± 0.0
38	137.132	C_10_H_17_^+^	Monoterpenes	49.6 ± 15.7	62.9 ± 12.4	82.3 ± 19.2	25.8 ± 5.8	39.6 ± 5.3	9.7 ± 3.5	5.1 ± 2.9	3.8 ± 1.2	1.3 ± 0.4	1.2 ± 0.6	2.4 ± 1.5
39	153.125	C_10_H_17_O^+^	Terpenoid-like compound	1.4 ± 0.4	1.1 ± 0.6	0.7 ± 0.5	0.0 ± 0.0	1.3 ± 0.7	0.7 ± 0.2	0.0 ± 0.0	0.0 ± 0.0	0.0 ± 0.0	0.0 ± 0.0	0.0 ± 0.0
40	155.142	C_10_H_19_O^+^	(E)-2-decenal	1.5 ± 0.7	1.1 ± 0.5	0.4 ± 0.4	0.0 ± 0.0	0.0 ± 0.0	0.0 ± 0.0	0.0 ± 0.0	0.0 ± 0.0	0.0 ± 0.0	0.0 ± 0.0	0.0 ±0.0
41	205.195	C_15_H_25_^+^	Sesquiterpenes	5.2 ± 3.5	1.8 ± 1.4	2.3 ± 2.9	1.5 ± 0.6	0.0 ± 0.0	0.0 ± 0.0	0.2 ± 0.1	0.0 ± 0.0	0.0 ± 0.0	0.0 ± 0.0	0.0 ± 0.0
Total VOC emissions				2981 ± 357	2170 ± 103	3072 ± 140	7587 ± 1347	3273 ± 368	3940 ± 368	3633 ± 408	3621 ± 294	4010 ± 756	3548 ± 568	8744 ± 636
Total Terpene emissions				873 ± 63	770 ± 62	740 ± 56	129 ± 22	226 ± 19	94 ± 14	66 ± 16	59 ± 7	42 ± 8	29 ± 8	13 ± 4
Total Ester emission				22 ± 6	24 ± 7	57 ± 17	125 ± 33	91 ± 20	113 ± 31	151 ± 35	150 ± 35	233 ± 77	324 ± 111	496 ± 99
Total Ripening-Linked VOCs				673 ± 77	772 ± 131	977 ± 112	4943 ± 1237	2098 ± 373	2853 ± 658	3779 ± 334	3088 ± 298	3237 ± 732	2855 ± 494	6049 ± 680

**Table 3:** PLS-DA statistics for each Y-Block (class 1 = ship; class 2 = truck; class 3 = plane) related to 66 mango samples. Sensitivity (SE); specificity (SP); class error, RMSEC, RMSECV, and RMSEP, R^2^ Cal. R^2^ CV, R^2^ Pred for calibration (Cal), cross-validation (CV), and prediction (Pred), respectively.

Statistics	Classes		
	Boat	Plane	Truck
**LVs**	3		
SE (Cal)	1.000	1.000	1.000
SP (Cal)	1.000	1.000	1.000
SE (CV)	1.000	1.000	1.000
SP (CV)	1.000	1.000	1.000
SE (P)	1.000	1.000	1.000
SP (P)	1.000	1.000	1.000
Class. Error (Cal)	0	0	0
Class. Error (CV)	0	0	0
Class. Error (Pred)	0	0	0
RMSEC	0.11	0.15	0.16
RMSECV	0.12	0.18	0.19
RMSEP	0.97	0.82	0.67
R^2^ Cal	0.93	0.91	0.84
R^2^ CV	0.93	0.87	0.77
R^2^ Pred	0.97	0.82	0.66

**Table 4:** Lists all VOCs with VIP scores > 1.2, tentatively identified as potential discriminants. The three *m*/*z* values with the highest VIP scores for each shipping class are highlighted in red.

Protonated *m*/*z*	Tentative Identification	VIP Scores		
		Boat	Truck	Plane
30.030	Ethylene (isotope)	0.83	1.9	0.85
43.018	Acetate fragment	0.74	1.39	0.49
45.033	Acetaldehyde	1.19	0.95	1.31
47.049	Ethanol	1.21	2.03	0.98
53.038	Fragments	1.22	X	X
81.069	Terpene fragments	1.25	0.75	1.27
91.054	Monoterpene fragments	1.17	0.47	1.23
93.069	Terpene fragments	1.21	0.24	1.26
95.086	Terpene fragments	1.22	0.92	1.31
99.080	(E)-2-hexenal	1.35	1.61	1.26
101.060	3-Hexen-1-ol	0.84	1.74	0.76
103.075	Methylbutanoic acid	1.21	0.45	1.28
117.091	Ethyl butanoate	0.98	0.43	1.26
121.101	Monoterpene fragments	1.04	1.45	0.89
123.117	Terpene fragments	1.05	1.64	0.86
135.117	Terpene, e.g., p-Cymene/a-Cedrene	0.92	1.35	0.79
137.132	Monoterpenes	1.29	0.98	1.33

**Table 5:** Results of the consumer evaluation by an untrained consumer (*n* = 65). Different capital letters indicate differences by the LSD test at the 99.0% confidence level (*p* = 0.01).

Mango Cultivar	Fruit Origin	Overall Judgment After Taste	Consumer Acceptability % (>5)
Tommy Atkins	Brazil	4.94 ± 1.1	0.40
Kent	Brazil	5.25 ± 1.1	0.52
Keitt	Brazil	6.02 ± 0.9	0.61
Osteen	Spain	7.03 ± 1.1	0.76
Kensington Pride	Italy	6.42 ± 0.6	0.73
Kent	Israel	6.88 ± 0.9	0.78
Kent	Brazil	7.20 ± 1.1	0.88
Palmer	Brazil	8.09 ± 1.3	0.94
Kent	Mexico	8.10 ± 1.2	0.92
Kent	Peru	8.37 ± 1.0	0.95
Sindhri	Pakistan	7.02 ± 1.2	0.72
By Boat		5.43 ± 0.5 A	0.51 ± 0.11 A
By Plane		7.61 ± 0.6 B	0.87 ± 0.09 C
By Truck		6.68 ± 0.4 B	0.75 ± 0.04 B

**Table 6:** Total GHG emissions, emissions intensity (kg CO_2_e/t·km), total distance (km), and transport modes for one complete route per country of origin, calculated using EcoTransIT World 2024 in accordance with the GLEC Framework v3.1.

Cultivars	Route	Mode	Total CO_2_e (kg)	Emissions Intensity (kg CO_2_e/t·km)	Distance Segment for Vehicles (km)
Kent, Palmer	Recife–Florence	Airplane + Truck	583,564	0.6315	7894
Keitt, Kent, Tommy Atkins	Recife–Florence	Sea ship + Truck	19,132	0.0185	8939
Kent	Piura–Florence	Airplane + Truck	1,010,918	0.7837	11,560
Kent	Guerreo–Florence	Airplane + Truck	772,664	0.6970	9750
Sindhri	Punjab–Florence	Airplane + Truck	485,053	0.6252	6316
Kent	Haifa–Florence	Airplane + Truck	358,388	0.7272	3591
Osteen	Granada–Florence	Truck	17,585	0.0913	1926
Kensington Pride	Palermo–Florence	Truck	9500	0.0916	1036

## Data Availability

The original contributions presented in this study are included in the article. Further inquiries can be directed to the corresponding author. The data are not publicly available due to privacy and ethical restrictions. The raw data supporting the conclusions of this article will be made available by the authors on request.
